# ABO, Lewis blood group systems and secretory status with H.pylori infection in yemeni dyspeptic patients: a cross- sectional study

**DOI:** 10.1186/s12879-023-08496-2

**Published:** 2023-08-08

**Authors:** Mohammed Abdulwahid Almorish, Boshra Al-absi, Ahmed M. E. Elkhalifa, Elham Elamin, Abozer Y. Elderdery, Abdulaziz H. Alhamidi

**Affiliations:** 1 Department of Hematology, Faculty of Medicine and Health Sciences, Sana’a University, Sana’a, Yemen; 2 Public Heath Department, College of Health Sciences, Saudi Electronic University, Riyadh, Saudi Arabia; 3 Department of Hematology, Faculty of Medical Laboratory Sciences, University of El Imam El Mahdi, Kosti, 1158 Sudan; 4 Department of Clinical Laboratory Sciences, College of Applied Medical Science, Jouf University, Sakaka, Saudi Arabia; 5 Clinical Laboratory Sciences Department, College of Applied Medical Science, King Saud University, Riyadh, Saudi Arabia

**Keywords:** H.pylori, ABO, Lewis, Le, Secretor

## Abstract

**Background:**

The ABO and Lewis blood group antigens are potential factors in susceptibility to H. pylori infection. This research aimed to examine the prevalence of Helicobater pylori (H.pylori) infection and its association with ABO, Lewis blood group systems, and secretory status in Yemeni symptomatic patients.

**Methods:**

In a cross-sectional study, 103 patients referred for endoscopy due to dyspepsia were included. H pylori infection was assessed using stool antigen and serum antibody rapid tests. ABO and Lewis blood group systems were examined using hemagglutination assay. Saliva samples were investigated for identification of the secretory phenotype using hemagglutination inhibition test.

**Results:**

The prevalence of H. pylori infection was (80.6%), with a higher rate of infection in females than males. The ABO blood groups were found to be significantly different between males and females (p = 0.047). The O blood group was prevalent among H. pylori patients, especially secretors. There was a significant association between ABO blood groups and H. pylori infection (p = 0.001). The Le (a + b+) phenotype was the most common, followed by Le (a + b-), Le (a-b+), and Le (a-b-). Lewis blood group systems and secretory status of symptomatic patients were not associated with H. pylori infection. The results showed that serum Ab test for H. pylori achieved poor sensitivity (68%), specificity of 55%; positive predictive value (PPV) 86%, negative predictive value (NPV) 29% and accuracy 65.1%.

**Conclusion:**

The prevalence of H. pylori infection was high in Yemeni patients. This infection was linked to the O and Le (a + b+) secretor phenotype. The H. pylori stool Ag test is the most reliable noninvasive diagnostic method for detecting H. pylori infection.

## Introduction

Human ABO antigens are located on the red cell surface, they play an active role in the cells’ physiology and pathology. They are oligosaccharide structures found on leukocytes, platelets, and tissues. Also, they are present in a soluble form in sweat, saliva, breast milk, and other body fluids [1, 2]. ABO (*ABO*), H (*FUT1*), secretor (*FUT2*) and Lewis (*FUT3*) blood system genes control the expression of the carbohydrate repertoire present in areas occupied by microorganisms [3]. The blood system genes play critical roles in the final ABO antigen structure of an individual’s body tissues and secretions [4]. The ABO, Lewis(Le), and Rhesus (Rh) blood group systems demonstrate how antigens can be classified into functional categories of structural proteins; enzymes; transporters and channels; adhesion molecules; and receptors for exogenous ligands, viruses, bacteria, and parasites [5].

Helicobacter pylori (H.pylori) is a helical, gram negative, microaerophilic bacterium, known to colonize the mucous membrane of the human stomach [6]. H. pylori is a major risk factor for chronic gastritis, peptic ulcers, and gastric cancer [7]. Globally, H pylori is a major gastric infection that is estimated to affect 50% of the population, and is present in both developed and developing countries [8]. Its prevalence is > 70% in developing countries [9, 10]. In contrast, the prevalence of H. pylori infection in Yemen is not well-defined, as various studies have reported a wide range of 10- 92.8% [11–13].

Blood group antigens can serve as receptors for lectins carried on the surface of various pathogens, facilitating invasion and colonization by binding microbial toxins and inducing infection [14]. Clinical data on the association between H. pylori, gastric cancer, and ABO/Le are contradictory. A higher incidence of H. pylori infection in O individuals and a lower incidence in A individuals have been reported [15]. Strikingly, the risk of developing peptic ulcers was higher in O individuals in a large population-based study [16]. The link between the Le and Se phenotypes seems to be even more unclear. For instance, Se status and H. pylori infection have been shown to be independent risk factors for gastric disease, with a higher risk in non-secretor patients [17], although Leb (Se) seems to play a crucial role in H. pylori adhesion [14]. At the tissue level, H. pylori-infected patients with gastric ulcers have increased Lea and loss of H and Leb expression in the inflamed gastric mucosa [18]. The blood-group antigen-binding adhesion A (BabA) and sialic acid–binding adhesion A (SabA) are important adhesins with carbohydrate-binding domains [19–22]. BabA binds to fucosylated glycoproteins carrying ABO/Le antigens, particularly H and Leb, which are expressed in gastric epithelial cells of secretors [23]. The tight adherence of BabA is believed to aid in the delivery of multiple virulence factors, such as VacA and CagA, that impact the signaling pathways of the mucosal epithelium [24]. SabA interacts with glycoconjugates containing sialyl-Lea and sialyl-Lex antigens, which are elevated during inflammation [25]. Therefore, the ability of H. pylori to initiate and maintain infection may be influenced by the regulation of BabA and SabA [26].

Our study aimed to determine the prevalence and the close association between ABO, Lewis blood groups, and secretory status and the validity of two non-invasive diagnostic tests in H. pylori infection.

## Methods

The study was conducted from August to December 2019 at the Department of Endoscopy and/or the Clinical Gastroenterology outpatient service of the Educational Republican Hospital in Sana’a City. One hundred and three adult patients were included in the study if they had symptoms of dyspepsia and upper gastrointestinal endoscopy was indicated. In our study Occult blood were examined to exclude gastric and duodenal bleeding ulcers (One Step Fecal Occult Blood Test Device; Abon Biopharm, China). Patients taking certain medications, such as H2-receptor antagonists, proton pump inhibitors, non steroidal anti-inflammatory drugs and antibiotics were excluded from the study if they had taken them within the past 4 weeks.

The calculation of the sample size in our study was based on the established prevalence rate of H. pylori, which was found to be 92.8% according to previous literature. With a margin of error or absolute precision of ± 5% and a confidence level of 95%, the sample size was determined to be 103. The present study employed the subsequent formula: N = Z^2^P(1 − P) /d^2^. This particular formula comprises the following variables: N designates the sample size, Z denotes the Z statistic for a given level of confidence (i.e., 1.96 for a 95% confidence level), P represents the expected prevalence or proportion, and d margin of error or precision (i.e., d = 0.05).

### H. pylori stool antigen test

H. pylori stool antigen tests were measured by using a one-step test device (ABON Bio Pharma, China) for the qualitative detection of H. pylori Ag in the feces. The one-step H. pylori stool Ag test device (ABON BioPharma, Hangzhou, China) is a chromatographic immunoassay for the qualitative detection of H. pylori antigen in human feces and provides results in 10 min with a sensitivity and specificity of > 99.9% [27].

Briefly, 50 mg (from formed stool) or two drops of liquid stool were transferred to a specimen collection tube containing extraction buffer. The tube was agitated vigorously by hand and left undisturbed for 2 min. Two drops of the extracted specimen were transferred to the specimen well on the test device, and the results were recorded after 10 min. According to the manufacturer’s instructions, the test is defined as positive if two distinct colored lines appear, and negative if one line appears.

### H. pylori serum antibody test

Serum samples were obtained by centrifugation at 3,000 rpm for 10 min.

For each serum sample, three drops were used to detect H. pylori antibodies using a rapid chromatographic immunoassay commercial kit (H. pylori One Step Test Device, DiaSpot H. pylori, Indonesia). This test qualitatively and selectively detects H. pylori antibodies in the serum or plasma by utilizing a combination of H. pylori antigen-coated particles and anti-human IgG. This test has a sensitivity > 95.9% and specificity of 75.9%, with an overall accuracy of 85.2%, compared to the culture/histology of endoscopic specimens for H. pylori [28]. The test was performed in accordance with the manufacturer’s instructions without any modifications. Briefly, three drops of the serum sample were applied directly to the sample well in the test device, and the results were read after 10 min. The appearance of one colored line in the control region indicated a negative result, whereas the appearance of two colored lines in the test region and control regions indicated a positive result.

### Expression of ABO and Rh antigens in blood

ABO and Rh blood group antigens were determined using standardized hemagglutination tests according to the manufacturer’s instructions with anti-A, anti-B, anti-AB and anti-D monoclonal antibodies (Lorne Labs. UK) [29].

### Expression of Lewis antigens in blood

Lewis blood group antigens were determined using standardized hemagglutination tests according to the manufacturer’s instructions with anti-Le^a^, and anti-Le^b^ monoclonal antibodies (Lorne Labs. UK) [29].

### Expression of A,B,H, Le^a^ and Le^b^ antigens in Saliva

The ABH and Lewis antigens in saliva were tested by hemagglutination inhibition.

methods with anti-A, anti-B, anti-H, anti-Le^a^, and anti-Le^b^ monoclonal antibodies (Lorne Labs. UK) [29].

### Ethical approval

This study was approved by the Research Ethics Committee of the Faculty of Medicine and Health Sciences, Sana’a University, Yemen. Written informed consent was obtained from all participants according to the Helsinki Declaration principles.

### Statistical analysis: statistical analysis

The generated data were coded, entered, validated, and analyzed using SPSS 23 (SPSS, Chicago, IL, USA). We tested for association in categorical variables using the chi-square test and reported the corresponding p-values. Sample means were compared using the Student’s t-test. Statistical significance was set at P < 0.05. Considering the stool Ag test as the gold standard, the sensitivity, specificity, positive predictive value (PPV), and negative predictive value (NPV) for serum antibody tests were calculated.

## Results

### Socio-Demographic and risk factors of H. pylori infection

A total of 103 patients were included in the study, with 40 (38.8%) being male and 63 (61.2%) female. The median age of H. pylori-infected patients was 25 years, with ages ranging from 19 to 57 years. The highest percentage of patients infected with H. pylori were under 30 years old, accounting for 70 (68%) of the total patients. The study also revealed that the prevalence of H. pylori was higher in rural areas (57.3%) compared to urban areas (42.7%). Interestingly, the study found that most patients obtained their water requirements from non-refined sources, with 62 (60.2%) patients falling under this category. Additionally, the majority of patients reported eating their meals at home routinely, with 82 (79.6%) patients indicating this. Furthermore, the distribution of ABO groups among patients showed that the O group was the most prevalent at 60.2%, followed by A at 33% and B at 6.8%. Additionally, 94 (91.3%) of patients were rhesus positive, while 92 (8.7%) were Rh negative. (Table 1)

**Table 1:** Socio-Demographic and Risk Factors of H. pylori Infection

Characteristics	No. of Patients (103)	
	№	%
**Sex**		
Male	40	38.8
Female	63	61.2
**Age**		
Median age (Min. - Max.)	25.0 (19.0–57.0)	
< 30	70	68
˃30	33	32
**Residence**		
Urban	44	42.7
Rural	59	57.3
**Drinking source**		
Non- Refined	62	60.2
Tap	41	39.8
**Eating source**		
Restaurant	21	20.4
Home	82	76.6
**ABO Blood groups**		
A	34	33
B	7	6.8
O	62	60.2
**Rh Typing**		
Positive	94	91.3
Negative	9	8.7

### Prevalence of H.pylori infection

**Fig. 1:**
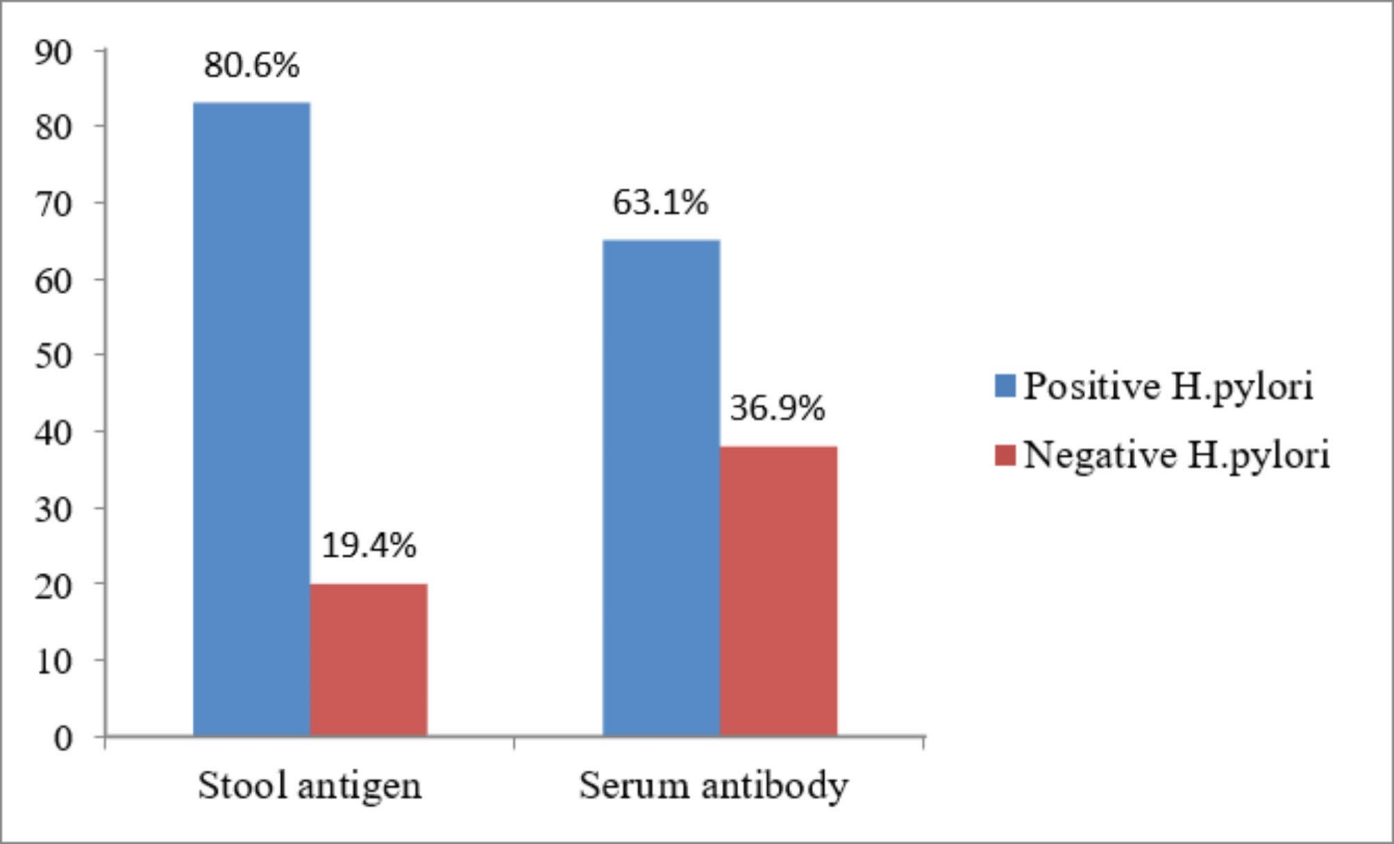
Prevalence of H. pylori infection according to stool antigen assay and serum antibody assay

The overall prevalence of H. pylori infection was 80.6% among symptomatic patients. Of the 103 patients examined using the H. pylori stool antigen assay, 83 (80.6%) and 20 (19.4%) patients were positive and negative, respectively. The number of positive stool Ag samples was higher in females 51 (49.5%) than in males 32 (31.1%). In the assay for detection of serum antibodies against H. pylori, there were 65(63.1%) seropositive cases, with 43 (41.8%) females and 22 (21.4%) males showing high and low seropositivity, respectively. The rate of H. pylori infection in female patients was not significantly higher than that in male patients using the two methods (p = 0.906; p = 0.178). (Table 2; Fig. 1)

**Table 2:** H. pylori stool antigen and serum antibody among the study patients (no = 103)

Variables		Sex		Total	P value
		Male №=40	Female №=63		
**Stool Ag**	**Positive**	32	51	83	0.906
	**Negative**	8	12	20	
**Serum Ab**	**Positive**	22	43	65	0.178
	**Negative**	18	20	38	
**Total**		40	63	103	

The sensitivity of the serum Ab test in relation to stool Ag test was 68% with a specificity of 55%, positive predictive value of 86%, and negative predictive value (NPV) 29%. The agreement between the two tests in the diagnosis of H. pylori infection was 65.1% (slight agreement) (Table 3). The antibody test failed to detect 27 H. pylori-positive samples using the antigen test. Nine samples were positive only for the antibody test. Test results were concordant for 56 samples. A total of 27 samples produced discordant results. (Table 3)

**Table 3:** Relation between results of H. pylori Serum antibody and Stool antigen among the study patients (no = 103)

Serum Ab.	Stool Ag							
	Positive N = 83	Negative N = 20	Total	Sensitivity %	Specificity%	PPV%	NPV%	Accuracy%
**Positive**	56	9	65	68	55	86	29	65.1
**Negative**	27	11	38					
**Total**	83	20	103					

Stool antigen test considered as gold standard

### Association of ABO, Rh phenotypes and secretory status with gender in patients with H.pylori infection

Out of the 103 H.pylori patients, 34 (33%) were blood group A; 7(6.8%) were blood group B and blood group O were 62 (60.2%). As regard the gender, the frequency of ABO blood groups in males was 28(27.2%) had group O, 8(7.8%) group A, 4(3.9%) group B and 0(0%) group AB. Similarly, of the 63 female patients, 34(33.0%) had group O, 26(25.2%) group A, 3(2.9%) group B and 0(0.0%) group AB. There was a statistically significant variation in the ABO blood groups between males and females and the prevalence blood group O was (34/63) higher in females than males(28/62) (p = 0.047) (Table 4).

**Table 4:** ABO, Rh phenotypes and secretor status among the gender in patients with H.pylori infection

Variables		Sex		Total	P value
		M	F		
**ABO**	**A**	8	26	34	0.047
	**B**	4	3	7	
	**O**	28	34	62	
**Rh**	**Positive**	36	58	94	0.721
	**Negative**	4	5	9	
**Secretor** **status**	**Secretor**	24	42	66	0.497
	**Non-secretor**	16	21	37	
**Total**		40	63	103	

A total of 94 (91.3%) of the patients were Rh positive and 9 (8.7%) of them were Rh negative (Table 1). The Rh positive blood group in the H.pylori patients were 36 (34.95%) males and 58 (56.3%) were females, while Rh negative H.pylori patients were 4 (3.88%) males and 5(4.85%) were females. Among the Rh positive patients, were 76(92%) H. pylori infected patients and 18 (90%) H. pylori non-infected patients. There was no statistically significant difference in the distributions of Rh phenotypes between gender of H. pylori patients (p = 0.721) (Table 4).

The frequency distribution of secretors and non-secretors across the gender of the study patients is presented in Table 4. In the study, 103 H.pylori patients representing 66 (64.1%)% and 37 (35.9%) were secretors and non-secretors, respectively. Among a total of 40 male patients, 24 (23.3%) were secretors and 16 (15.5%) were non-secretors. The 63 female patients had 42 (40.8%) secretors and 21 (20,4%) non-secretors among them. However, the difference between gender and secretor status was not statistically different (p = 0.497).

### Association of ABO, Lewis blood group phenotypes and secretor status in patients infected by H. pylori

Regarding the ABO blood group in H. pylori infection, the distribution of H. pylori infected patients was 42 (51%) in blood group O, followed by blood group A in 34 (41%), blood group B in 7 (8%), and the distribution of non-infected patients by H. pylori was 20 (100%) observed only in blood group O. There was a significant association between the ABO blood group and infected and non-infected patients (p = 0.001).

Among a total of 83 H.pylori infected patients, 52 (63%) were secretors and 31 (37%) were non- secretors and a total of 20 H.pylori non-infected patients had 14 (70%) secretors and 6 (30%) non-secretors. There was non-significant association between secretor status and infected and non-infectedpatients with H. pylori and using noninvasive immunochromatographic Stool Ag assay (p = 0.367). (Table 5)

**Table 5:** ABO and Lewis blood group phenotypes and secretor status between infected and non-infected patients by H. pylori. (n = 103)

Variables	Positive Stool Ag (Infected)		Negative Stool Ag (Non-infected)		Total		*P* value
	**N**	**%**	**N**	**%**	**N**	***%***	
**ABO phenotypes**:							
A	34	41	0	0	34	33	**0.001**
B	7	8	0	0	7	6.8	
O	42	51	20	100	62	60.2	
**Lewis phenotypes**							
**Secretor**							
Le (a-b+)	17	20	5	25	22	21.4	0.807
Le (a + b+)	35	42	9	45	44	42.7	
**Non-secretor**							
Le (a + b-)	28	34	6	30	34	33	
Le (a-b-)	3	4	0	0	3	2.9	
**Secretion status**							
Secretor	52	63	14	70	66	64.1	0.367
Non-secretor	31	37	6	30	37	35.9	
**Rh**							
**Positive**	76	91.6	18	90	94	91.3	0.824
**Negative**	7	8.4	2	10	9	8.7	

The study reported secretor status belong to Lewis Le (a-b+), Le (a + b+) and those who are non- secretors to Le (a-b-) and Le (a + b-) blood group phonotype. The most common phenotype in the Lewis groups was Le (a + b+) 44(42.7%) followed by Le (a + b-) 34 (33%), Le (a-b+) 22(21.4%) and Le (a-b-) 3 (2.9%). Among a total of 83 H.pylori infeccted patients, 52 (63%) were secretors; where Le (a-b+) was 17(20%) and Le (a + b+) was 35(42%) and 31(37%) were non-secretors; where, Le (a + b-) was 28(34%) and Le (a-b-) was 3(4%). Among a total of 20 H.pylori non infeccted patients, where Le (a-b+) was 5 (25%) and Le (a + b+) was 9(45%) and 6 (30%) were non-secretors of Le (a + b-) phenotype. (Table 5)

As regard the secretory status in saliva with ABO blood groups in H.pylori infection, 66 (64.1%) were secretors, where, the blood group O was 40 (60.6%) followed by blood groups A patients 22 (33.4%) and blood group B 4 (6.1%). However, 37 (35.9%) of non-secretors, the blood group O was 22 (59.5%) followed by blood groups A patients 12 (32.4%) and blood group B 3 (8.1%). Data show that there was no association between secretor status and ABO blood groups in patients infected by H. pylori (p = 0.924) (Table 6).

**Table 6:** ABO blood group between Lewis blood group phenotypes and secretor status in patients with H.pylori infection

Variables		ABO blood group			Total	*P value*
		A	B	O		
**Secretor status**	**Secretor** **Non-secretor**	22	4	40	66	**0.924**
		12	3	22	37	
**Lewis phenotypes** **Secretor** **Le (a-b+)** **Le (a + b+)** **Non-secretor** **Le (a + b-)** **Le (a-b-)**						**0.671**
		5	1	16	22	
		17	3	24	44	
		10	3	21	34	
		2	0	1	3	
**Total**		34	7	62	103	

The link between the Lewis blood group and infected and non-infected patients was not significant (p = 0.807), and there was no correlation between the Lewis blood group and ABO in H. pylori patients (p = 0.671).

## Discussion

The prevalence of H. pylori infection in Yemen was found to be high at 80.6%, which is similar to other countries in the Middle East and North Africa region [30]. However, a recent study from Yemen found a lower rate of infection at 19.3%, which could be due to factors such as socioeconomic status and living conditions [31]. No previous study has been conducted on ABO, Lewis group systems, and secretory status in H. pylori-infected patients in Yemen.

In this study, the rate of H. pylori infection in females is not significantly different than in males. Some studies show higher rates in females than males, while others show higher rates in men, but overall there is no significant relationship between gender and H. pylori infection [31–33]. Studies show that males may have a higher rate of H. pylori infection but in general, there is no significant relationship between sex and H. pylori infection rate. This implies due to hormonal differences between sex and has no role in the H. pylori infection [34–37].

The results of our study found that H. pylori infection is most prevalent among patients under 30 years old, with a significant association between infection rate and age group. This finding has been supported by studies in Iraq and Iran [38, 39]. However, other studies have shown high infection rates in the 43-50-year-old age group, but this difference is not significant [40, 41]. A previous studies observed that H. pylori infection is more common in people under 50 years old and may be involved in the development of Colorectal Adenomatous Polyps [42, 43].The age group under 30 is particularly vulnerable to infectious diseases due to their active lifestyle and potential lack of personal hygiene and healthcare. H. pylori colonization starts at a young age and exposure to multiple sources of infection increases with age [43–47].

This study found that the prevalence of H. pylori, a bacteria that can cause stomach ulcers and cancer, was slightly higher in rural areas compared to urban areas, but this was not statistically significant. This finding agrees with studies from Yemen and Iraq [31, 44] but disagrees with studies from Tanzania and China [45, 46].The variation in prevalence could be due to factors such as poor water supply, inadequate sewage disposal, social habits, and low education levels among low-income populations [48, 49].

Our data revealed that the serum antibody test displayed a sensitivity of 68% compare to the stool antigen test, with a specificity of 55%. Additionally, it exhibited a PPVof 86%, while its NPV was noted to be 29%. A study conducted in Yemen revealed that the serum antibody test had a sensitivity of 50% and specificity of 65%. Additionally, the PPV was 65% and the NPV was 50% [47].

Abadi and colleagues discuss the benefits of stool antigen testing, including its accuracy, ease of use, and popularity. However, the test is limited by factors such as bleeding, antibiotic use, bowel movements, and proton pump inhibitors. The authors recommend using monoclonal antibodies to measure and eliminate H. pylori, as well as for initial screening in clinical settings [50]. The stool antigen test is a reliable diagnostic tool for H. pylori according to numerous studies. It has been compared to gold standard methods like breath test and biopsy bacterial culture. For small laboratories lacking advanced equipment, the test has been proposed as a useful alternative [50–54].

The present study observed that a significant association between H. pylori infection and ABO blood group, with patients who have blood group O being more susceptible to the H.pylori infection. A meta-analysis study suggests that O blood type may be a risk factor for H. pylori infection [55].Additionally, the study confirms that O blood type increases the risk of H. pylori infection while A/AB blood type is associated with a predisposition to gastric cancer [56].

The ABO and Lewis histo-group antigens may affect susceptibility to H.pylori infection [3]. In our investigation, the most H. pylori patients were found to be O secretors, but there was no significant difference between secretor status in infected and non-infected patients. A recent study found that non-secretors were more prone to H. pylori infections,[7] but another study found that secretors were at a higher risk in H.pylori infection [57]. The bacteria may attach to the Lewis b antigen, which is expressed on the surface of the gastric mucosa also correlates with infectious disease risk [58], and non-secretors may be resistant to H. pylori [59]. H. pylori have a protein called BabA that binds to a type 1 H antigen, which are commonly found in the stomach lining, [60] and able to attach to Leb, which is found in high levels in stomach cells related to O and secretor phenotype [62]. This explains why people with type O blood are more prone to gastrointestinal diseases like gastritis and stomach ulcers.

Different studies have found varying prevalence of Lewis blood group phenotypes [60–63]. Our study found Le (a + b+) to be the most common phenotype. A meta-analysis reported that secretors often have the Le (a-b+) phenotype, while non-secretors have Le (a-b-) and different secretor status and their phenotypes vary in prevalence in different populations. The secretor phenotype is present in all populations but more prevalent in Caucasians, whereas the Le (a + b-) phenotype is found in over 20% of Caucasians and Blacks but is rare in Asians [7]. In two similar studies found Le (a- b+) to be most prevalent [62, 63]. The Le(a-b+) phenotype is frequent in all populations, while the Le (a + b+) phenotype is more common in Asians and Polynesians. Lastly, the Le(a-b-) phenotype is rare in Caucasians but common among Blacks [63, 64]. The cause for these differences is unclear, but it is thought that disease-causing microorganisms may have a role in this process [15].

Data from this study showed a significant association between ABO blood groups, but not between Lewis and secretor phenotypes and H. pylori infection. Previous studies have shown an association between antigens of these groups with susceptibility to H. pylori colonization [14, 15]. Recent research suggests that these blood group systems can affect susceptibility to infection, disease progression, and immune response [26].

## Conclusion

Based on our findings, it is important to consider the blood group of individuals when assessing their risk for H. pylori infection. Those with O blood may be more susceptible to infection due to their increased likelihood of being secretors. Additionally, the presence of the Le (a + b+) phenotype may also increase an individual’s risk for H. pylori infection. To effectively screen for H. pylori infection, we recommend using the sensitive H. pylori stool Ag test as a non-invasive screening method before resorting to invasive procedures such as endoscopy or biopsy. This approach could help identify infected individuals earlier and potentially prevent further complications associated with untreated H. pylori infections.

## Data Availability

The datasets used and analyzed during the current study are available from the corresponding author on reasonable request.
