# Correction: ABO, Lewis blood group systems and secretory status with H. pylori infection in yemeni dyspeptic patients: a cross-sectional study

**DOI:** 10.1186/s12879-023-08622-0

**Published:** 2024-01-15

**Authors:** Mohammed Abdulwahid Almorish, Boshra Al-absi, Ahmed M. E. Elkhalifa, Elham Elamin, Abozer Y. Elderdery, Abdulaziz H. Alhamidi

**Affiliations:** 1 Department of Hematology, Faculty of Medicine and Health Sciences, Sana’a University, Sana’a, Yemen; 2 Public Heath Department, College of Health Sciences, Saudi Electronic University, Riyadh, Saudi Arabia; 3 Department of Hematology, Faculty of Medical Laboratory Sciences, University of El Imam El Mahdi, 1158 Kosti, Sudan; 4 Department of Clinical Laboratory Sciences, College of Applied Medical Science, Jouf University, Sakaka, Saudi Arabia; 5 Clinical Laboratory Sciences Department, College of Applied Medical Science, King Saud University, Riyadh, Saudi Arabia

**BMC Infectious Diseases (2023) 23:520**

10.1186/s12879-023-08496-2

The original publication of this article contained an incorrect author name. The incorrect and correct information is listed in this correction article. The original article has been updated.

Incorrect:

Abozer Y Eldedery

Correct:

Abozer Y Elderdery

